# Implementation of a human papillomavirus vaccination demonstration project in Malawi: successes and challenges

**DOI:** 10.1186/s12889-017-4526-y

**Published:** 2017-06-26

**Authors:** Kelias Phiri Msyamboza, Beatrice Matanje Mwagomba, Moussa Valle, Hastings Chiumia, Twambilire Phiri

**Affiliations:** 1 World Health Organization, Malawi Country Office, ADL House, City Centre, P.O. Box 30390, Lilongwe, Malawi; 2 Public Health and Family Medicine Department, University of Malawi, College of Medicine, Lilongwe, Malawi; 3 Ministry of Health, Non-communicable diseases and Mental Health Unit, Lilongwe, Malawi; 4 Global Health Implementation Program, School of Medicine, University of St Andrews, St Andrews, Scotland UK; 5 Ministry of Health, Expanded Programme for Immunization (EPI) Unit, Lilongwe, Malawi; 6 Ministry of Health, Reproductive Health Directorate, Lilongwe, Malawi

**Keywords:** Cervical cancer, Human papilloma virus, HPV vaccination, Sub-Saharan Africa, Malawi

## Abstract

**Background:**

Cervical cancer is a major public health problem in Malawi. The age-standardized incidence and mortality rates are estimated to be 75.9 and 49.8 per 100,000 population, respectively. The availability of the human papillomavirus (HPV) vaccine presents an opportunity to reduce the morbidity and mortality associated with cervical cancer. In 2013, the country introduced a school-class-based HPV vaccination pilot project in two districts. The aim of this study was to evaluate HPV vaccine coverage, lessons learnt and challenges identified during the first three years of implementation.

**Methods:**

This was an evaluation of the HPV vaccination project targeting adolescent girls aged 9–13 years conducted in Malawi from 2013 to 2016. We analysed programme data, supportive supervision reports and minutes of National HPV Task Force meetings to determine HPV vaccine coverage, reasons for partial or no vaccination and challenges. Administrative coverage was validated using a community-based coverage survey.

**Results:**

A total of 26,766 in-school adolescent girls were fully vaccinated in the two pilot districts during the first three years of the programme. Of these; 2051 (7.7%) were under the age of 9 years, 884 (3.3%) were over the age of 13 years, and 23,831 (89.0%) were aged 9–13 years (the recommended age group). Of the 765 out-of-school adolescent girls aged 9–13 who were identified during the period, only 403 (52.7%) were fully vaccinated. In Zomba district, the coverage rates of fully vaccinated were 84.7%, 87.6% and 83.3% in year 1, year 2 and year 3 of the project, respectively. The overall coverage for the first three years was 82.7%, and the dropout rate was 7.7%. In Rumphi district, the rates of fully vaccinated coverage were 90.2% and 96.2% in year 1 and year 2, respectively, while the overall coverage was 91.3%, and the dropout rate was 4.9%. Administrative (facility-based) coverage for the first year was validated using a community-based cluster coverage survey. The majority of the coverage results were statistically similar, except for in Rumphi district, where community-based 3-dose coverage was higher than the corresponding administrative-coverage (94.2% vs 90.2%, *p* < 0.05), and overall (in both districts), facility-based 1-dose coverage was higher than the corresponding community-based (94.6% vs 92.6%, *p* < 0.05). Transferring out of the district, dropping out of school and refusal were some of the reasons for partial or no uptake of the vaccine.

**Conclusion:**

In Malawi, the implementation of a school-class-based HPV vaccination strategy was feasible and produced high (>80%) coverage. However, this strategy may be associated with the vaccination of under- and over-aged adolescent girls who are outside of the vaccine manufacturer’s stipulated age group (9–13 years). The health facility-based coverage for out-of-school adolescent girls produced low coverage, with only half of the target population being fully vaccinated. These findings highlight the need to assess the immunogenicity associated with the administration of a two-dose schedule to adolescent girls younger or older than 9–13 years and effectiveness of health facility-based strategy before rolling out the programme.

## Background

Globally, cervical cancer has been identified as the fourth most common cancer in women (after breast, colorectum and lung), with an estimated 528,000 new cases and 266,000 deaths occurring each year. The majority (>85%) of global cervical cancer morbidity and mortality occur in developing countries and the highest risk is in eastern and southern Africa [1, 2]. Virtually all cervical cancer cases (99%) are caused by human papilloma virus (HPV), with HPV types 16 and 18 causing 70% of the cases and precancerous lesions. HPV is transmitted through sexual contact and is the most common viral infection of the reproductive tract [3, 4]. Currently, there are two WHO prequalified vaccines that protect against both HPV type 16 and 18. One of the vaccines also protects against HPV types 6 and 11, which cause anogenital warts. HPV vaccines are administered before the onset of sexual activity, i.e., before exposure to HPV infection. For quadrivalent HPV vaccine, WHO recommends a 2-dose schedule (0.5 ml at 0 and 6 months) for adolescents aged 9–13 years and a 3-dose schedule (0.5 ml at 0, 1, 6 months) for those aged 14 years or more. For bivalent vaccine, 2-dose schedule is recommended for the age group of 9–14 years and a 3-dose schedule for those aged 15 years or more [5].

In Malawi, cervical cancer is a major public health problem. The age-standardized incidence and mortality rates (ASRs) are estimated to be 75.9 and 49.8 per 100,000 population, respectively. It is the commonest cancer in women accounting for 45.4% of all cancers and the trend is increasing. It is estimated that every year, 3684 women develop cervical cancer, and 2314 women die from the disease [6–9]. The median and 5-year survival rates from the time of diagnosis have been reported to be 10 months and 2.9%, respectively [10]. The high prevalence of HPV (33.6%), co-infection with HIV (10.8%) and inadequate screening and treatment services for precancerous lesions have been identified as the main risk factors for high cervical cancer incidence and mortality. Overall, Malawi has a population of 4.76 million women aged 15 years and older who are at risk of developing cervical cancer [8, 9, 11, 12].

Ministry of Health through its Reproductive Health (RH) Directorate has been implementing a national cervical cancer screen-and-treat programme using visual inspection with acetic acid (VIA) and cryotherapy since 2004 [13]. In 2013, with support from the Global Alliance for Vaccines and Immunization (GAVI), the country introduced HPV vaccination demonstration project. This study aimed to evaluate HPV vaccine coverage, lessons learnt and challenges identified during the first three years of implementation.

## Methods

### HPV vaccination delivery strategy

For in-school adolescent girls, school-class-based strategy was implemented. For out-of- school, a health facility-based strategy was used. According to data from the education management information system (EMIH), majority (>85%) of girls aged 9–13 years are in standard/grade 4. Standard 4 was therefore selected, and all the girls in that class irrespective of age were eligible to receive the vaccine. Out-of-school girls aged 9–13 years were identified and registered in the communities by community health workers with support from the community chiefs. The parents or caregivers of out-of-school girls were advised to take them to the nearest health facility during the vaccination week.

### Vaccine type and administration

Malawi successfully applied to GAVI and sourced Gardasil® (Merck & Co., USA); a quadrivalent HPV vaccine that prevents infections from the four HPV genotypes (6, 11 and 16, 18) that are the most common cause of cervical cancer (HPV types 16 and 18) and genital warts (HPV types 6 and 11) [3–5]. The vaccines were administered by health surveillance assistants (HSAs), who are government-employed community health workers who administer routine expanded programme of immunization (EPI) vaccines and other preventive and disease surveillance interventions. At the school, there was an HPV registry for all girls in standard 4 and their HPV vaccination status. Each girl was given an HPV vaccination card on which doses and date given were recorded.

The intervention was planned and supervised by three sections of the Ministry of Health – the Non-communicable Diseases (NCDs) Unit, the EPI Unit and the RH Department – in collaboration with the Ministry of Education School Health and Nutrition Department and School Inspection Unit. Technical support was provided by WHO, UNICEF, CHAI and PATH.

### Demonstration districts

Rumphi district, located in northern region, was selected to represent a rural district, while the Zomba district, located in the southern region, was chosen to represent both urban and rural settings.

### Study type

This was a cross-sectional study with two arms: facility- and community-based with the aim of documenting administrative- and community- based vaccine coverage, respectively. The facility-based arm was evaluated by compiling and analysing data from schools and health facilities in the two implementing districts and reviewing supportive supervision reports and minutes of National Task Force meetings.

To validate administrative vaccine coverage and identify reasons for partial or no vaccination, a community-based vaccine coverage survey was conducted. WHO immunization cluster survey guidelines (popularly known as the 30 by 10 method) were used to randomly select 30 clusters (enumeration areas (EA)) from each of the two districts using the probability proportional to size (PPS) sampling method. In each cluster, 10 eligible households with a girl in standard 4 or out- of- school aged 9–13 years were selected using a systematic sampling method. Data were collected using a standardized structured questionnaire. The questionnaire was designed to collect information on the demographic characteristics of the eligible girl and her caregiver; status of HPV immunization, including the dates and location where the vaccine was received; reasons for vaccine acceptance or non-acceptance; exposure to information from education and communication (IEC) materials and messages; and knowledge and perceptions of the HPV vaccine. Verbal reports from parents or caregivers regarding the vaccination status of the girls were verified using vaccination cards, where available, or the school’s HPV registry.

### Data management

Data were entered into a Microsoft Excel® spreadsheet and exported to Epi Info version 7 (Centers for Disease Control, Atlanta, GA, USA) and SPSS for Windows version 20 (Chicago, IL, USA) for analysis. Chi-square tests were used to evaluate differences between administrative and community- based coverage and drop- out rates between the 1st and 2nd dose. All analyses were performed using 95% confidence levels, and *p* < 0.05 was considered statistically significant.

## Results

### Characteristics of adolescent girls enrolled in the HPV vaccination demonstration project 2013–2016

A total of 26,766 in-school adolescent girls were fully vaccinated in the two pilot districts during the first three years of the project. Of these; 2051 (7.7%) were under the age of 9 years, 884 (3.3%) were over the age of 13 years, and 23,831 (89.0%) were aged 9–13 years (the recommended age group). Of the 765 out-of-school adolescent girls aged 9–13, only 403 (52.7%) were fully vaccinated (Table 1).

**Table 1:** Age of standard/grade four girls who received HPV vaccine

	Year 1 (2013–2014)			Year 2 (2014–2015)			Year 3 (2015–2016)			Total for the first 3 years of the project		
	Zomba urban (%)	Rumphi District (%)	Total (%)	Zomba urban (%)	Rumphi District (%)	Total (%)	Zomba district (%)	Rumphi District (%)	Total (%)	Zomba district (%)	Rumphi District (%)	Total (%)
Total in-school standard 4 girls fully vaccinated:	1635	3874	5509	1506	3055	4561	13,532	3164	16,696	16,673	10,093	26,766
≤8 years old	377 (23.1)	267 (6.9)	644 (11.7)	294 (19.5)	223 (7.3)	517 (11.3)	695 (5.1)	195 (6.2)	890 (5.2)	1366 (8.2)	685 (6.8)	2051 (7.7)
9–13 years old	1241 (75.9)	3484 (89.9)	4725 (85.8)	1176 (78.1)	2760 (90.3)	3936 (86.3)	12,252 (90.5)	2918 (92.2)	15,170 (91.0)	14,669 (88.0)	9162 (90.8)	23,831 (89.0)
≥14	17 (1.0)	123 (3.2)	140 (2.5)	36 (2.4)	72 (2.4)	108 (2.4)	585 (4.3)	51 (1.6)	636 (3.8)	638 (3.8)	246 (2.3)	884 (3.3)
Out-of-school girls aged 9–13 years vaccinated	-	-	-	4/4 (100.0)	28/58 (48.3)	32/62 (51.6)	368/701 (52.5)	3/3 (100.0)	371/704 (52.7)	372/705 (52.8)	31/60 (51.7)	403/765 (52.7)
Total number of girls fully vaccinated	1635	3874	5509	1510	3083	4593	13,900	3167	17,067	17,045	10,124	27,169

In year one, fully vaccinated was defined receiving all the three (3) doses given at 0,2 and 6 months. In year 2 and 3, fully vaccinated was defined receiving two doses given at 0 and 6 months

### HPV vaccination coverage

In Zomba district, the coverage rates of fully vaccinated were 84.7%, 87.6% and 83.3% in year 1, year 2 and year 3 of the project, respectively. The overall coverage for the first three years was 82.7%, and the dropout rate was 7.7%. In Rumphi district, the coverage rates of fully vaccinated were 90.2% and 96.2% in year 1 and year 2, respectively, and the overall coverage and dropout rates were 91.3% and 4.9%, respectively. In Rumphi, 2nd dose was not administered during year 3 (Table 2). The administrative (facility-based) coverage for the first year was validated based on community-based coverage. The majority of the 3-dose coverage rates were statistically similar, except for in the Rumphi district, where community-based coverage was higher than administrative-coverage (94.2% vs 90.2%, *p* < 0.05), and overall (both district), facility-based 1-dose coverage was higher than community-based coverage (94.6% vs 92.6%, *p* < 0.05). The reasons for these differences were unknown (Table 3).

**Table 2:** School-class-based HPV coverage for the first three years of the project

	Year 1 (2013–2014)			Year 2 (2014–2015)		Year 3 (2015–2016)		Overall; the first 2 or 3 years of the project	
	1 Dose	2 Doses	3 Doses	1 Dose	2 Doses	1 Dose	2 Doses	1 Dose	Fully vaccinated
Zomba:									
Target	1930	1930	1930	1720	1720	16,531	16,531	20,181	20,181
No. vaccinated	1760	1675	1635	1575	1506	14,911	13,532	18,246	16,673
Coverage	91.2%	86.8%	84.7%	91.6%	87.6%	90.2%	83.3%	90.4%	82.7%
Dropout rate	ref	4.4%	6.5%	ref	4.0%	ref	6.9%	ref	7.7%
Rumphi:									
Target	4294	4294	4294	3296	3296	3303	3303	7590	7590^a^
No. vaccinated	4130	4002	3874	3169	3055	3167	-	7299	6929
Coverage	96.2%	93.2%	90.2%	96.2%	92.7%	95.9%	-	96.2%	91.3%
Dropout rate	ref	3.0%	6.0%	ref	3.5%	ref	-	ref	4.9%
Both districts:									
Target	6224	6224	6224	5016	5016	19,834	19,834	11,240	11,240^a^
No. vaccinated	5890	5677	5509	4744	4561	18,078	-	10,634	10,070
Coverage	94.6%	91.2%	88.5%	94.6%	90.9%	91.2%	-	94.6%	89.6%
Dropout rate	ref	3.4%	6.1%	ref	3.7%	ref		ref	5.0%

*Ref* reference, ^a^In Rumphi results for fully vaccinated were for the first two years

In year one, fully vaccinated was defined receiving all the three (3) doses given at 0, 2 and 6 months. In year 2 and 3, fully vaccinated was defined receiving two doses given at 0 and 6 months

**Table 3:** Validation of Year 1 HPV Vaccination administrative vs community coverage survey

	Facility-based coverage			Community-based coverage			
	Target (n)	Coverage (%)	95% CI	n	Coverage (%)	95% CI	*p* value
Zomba:							
1 dose	1930	91.2	89.9–92.5	309	87.7	83.3–92.1	0.05
2 doses	1930	86.8	85.3–88.3	309	87.4	82.9–91.8	0.78
3 doses	1930	84.7	83.1–86.3	309	85.8	81.3–90.3	0.63
Rumphi							
1 dose	4294	96.2	95.6–96.8	307	97.4	95.6–99.2	0.30
2 doses	4294	93.2	92.4–94.0	307	95.8	93.9–97.8	0.09
3 doses	4294	90.2	89.3–91.1	307	94.2^a^	91.7–96.7	0.03
Both districts							
1 dose	6224	94.6^a^	94.0–95.2	616	92.6	89.9–95.3	0.03
2 doses	6224	91.2	90.5–91.5	616	91.6	89.0–94.2	0.77
3 doses	6224	88.5	87.7–89.3	616	90.0	87.2–92.7	0.29

*CI* confidence interval, % percentage, ^a^statistically significant facility vs community based coverage

### Reasons for no or partial vaccination

The analysis of 65 in-school adolescent girls in Zomba who did not receive all the doses revealed that no or partial vaccination coverage occurred because of the following as reasons: 50 (76.9%) girls were transferred out of the district, 7 (10.8%) girls dropped out of school, 6 (9.2%) girls refused (of whom 5 were self-refusal and 1 was a parental refusal) and 2 (3.1%) girls were absent during the time when the 2nd dose was administered.

Community-based data from 81 parents/caregivers of the adolescent girls who did not complete the three doses in year one of the project revealed that refusal to be vaccinated by the girl, inconvenient location/time, and parent/caregiver beliefs that the vaccine was not good for the girls and that the vaccination site was unclean and/or unsafe were the most common reasons for partial or no uptake of the vaccine, accounting for 26.2%, 19.1%, 11.9% and 9.5% of refusals, respectively (Table 4).

**Table 4:** Reasons (%) for no or partial vaccination cited by care givers of un- or partially vaccinated eligible girls

Reason	Rumphi (*n* = 23)	Zomba urban (*n* = 58)	Overall (*N* = 81)
Girl not at risk for cervical cancer	20.0	5.4	7.1
Does not believe vaccination is good for child	0.0	13.5	11.9
Girl didn’t want to be vaccinated	40.0	24.3	26.2
Vaccination venue was unclean and unsafe	20.0	8.1	9.5
Location/time was inconvenient	0.0	21.6	19.1
Someone else said vaccine not good idea	0.0	5.4	4.8
Others in community or school were also refusing	0.0	2.7	2.4
Waiting time was unacceptable	0.0	5.4	4.8
Girls are too young for HPV vaccine	0.0	5.4	4.8
Was not aware of HPV vaccine program	20.0	5.4	7.1
Too much pain after 1st or 2nd dose	0.0	2.7	2.4
Girl ill on vaccination day	0.0	8.1	7.1
Girl absent from school on vaccination day	20.0	2.7	4.8

*n* number of respondents by district, *N* total number of respondents in both districts

## Discussion

This study showed that it was feasible and acceptable to administer HPV vaccine to adolescent girls using school-class-based strategy in Malawi. High coverage of fully immunized (at least 2 doses) of 86.5%, 91.1% and 83.3% were achieved in year 1, 2 and 3, respectively.

Cumulatively, at least 26,766 in-school adolescent girls were fully vaccinated during the first 3 years of the project High immunization coverage rates using school-class-based strategy was also reported in Tanzania (83.8%), Uganda (87.8%) and Rwanda (93.2%). Strong coordination between health and education officials, designing delivery strategy based on a good understanding of the current system and opportunities for synergy, active involvement of teachers, implementation through the regular EPI system structure, human resources, and government endorsement and ownership of the programme have been reported to be factors that facilitate the achievement of high rates of HPV vaccine coverage using school-based programmes [14–16]. In addition, compared with health facility-based strategies, the use of school-based strategies has been found to reduce operational problems for parents or caregivers [17].

The other lesson learnt from this study was that the school-class-based strategy is likely to include girls outside the vaccine manufacturers’ stipulated age group of 9–13 years. Of the 26,766 girls that were fully vaccinated; 2051 (7.7%) and 884 (3.3%) were aged less than 9 and more than 13 years, respectively. Up to 21% of standard 4 girls in urban schools were aged less than 9 years, whereas those aged 14 years and older were mainly identified in rural areas. Safety and efficacy data regarding the vaccination of girls aged 8 years or less are not readily available. Nevertheless, no serious adverse event following immunization was reported in general and in girls aged 8 years or less or 14 or more in particular. For girls aged 14 years or older, the vaccine manufacturer stipulates that a 3-dose (0.5 mL at 0, 2, 6 months) schedule should be administered for quadrivalent HPV vaccine. However, when using a class-based strategy, it might not be feasible to administer 3 doses to some girls while the rest receive 2 doses. Therefore, when the decision was made to change from a 3- to 2-dose schedule, all the girls in standard/grade 4 received a 2-dose schedule regardless of age. It was not known whether immunogenicity of those aged 8 years and less or 14 years and older were similar or inferior to those aged 9–13 years.

Most countries in southern Africa implemented school-based vaccination strategy during the HPV demonstration project. Therefore, data collected based on health facility-based strategy is scarce. Our demonstration project included a sizeable number of out-of-school girls, particularly when the project was scaled-up to the whole district of Zomba, who were vaccinated using a health facility-based strategy and, therefore, provided some insight on the coverage associated with the application of this strategy. Of the 765 of-out-school girls who were identified and parents/caregivers were advised to take them to the nearest health facility for vaccination, only half (53%) were fully vaccinated. This result occurred despite intensified community mobilization campaign.

Although the low coverage of out-of-school girls could not be generalized to in-school girls; this finding raised some fears that the health facility-based strategy in this population may not produce intended effective (>80%) coverage. Health facility- and child health day-based strategies (other than the school strategy) in Cameroon and Uganda also resulted in low coverage, ranging from 50% to 60% [18–20].

## Conclusion

In Malawi, the implementation of a school-class-based HPV vaccination strategy was feasible and resulted in a high (>80%) coverage. However, the school- class-based strategy may be associated with the vaccination of under- and over-aged girls who are outside the vaccine manufacturer’s stipulated age group of 9–13 years. The health facility-based coverage for out-of-school girls resulted in a low coverage, with only half of the target population being fully vaccinated. Our findings highlight the need to assess the immunogenicity associated with the administration of a two- dose schedule to adolescent girls aged younger or older than 9–13 years and effectiveness of health facility-based strategies before rolling out.
